# Zinc inhibits the reproductive toxicity of Zearalenone in immortalized murine ovarian granular KK-1 cells

**DOI:** 10.1038/srep14277

**Published:** 2015-09-23

**Authors:** Yijia Li, Xiaoyun He, Xuan Yang, Kunlun Huang, Yunbo Luo, Liye Zhu, Yuzhe Li, Wentao Xu

**Affiliations:** 1 College of Food Science and Nutritional Engineering, China Agricultural University, Beijing, China, 100083; 2 The Supervision, Inspection and Testing Center of Genetically Modified Organisms, Ministry of Agriculture, Beijing, China, 100083

## Abstract

Zearalenone (ZEA) mainly injures the reproductive system of mammals. In the present study, we aimed to explore the mechanism by which zinc inhibits ZEA-induced reproductive damage in KK-1 cells for the first time. The results shown that both zinc sulfate and zinc gluconate addition increased the intracellular zinc concentration and influenced the expression of zinc transporters (Slc30a1 and Slc39a1) in a time-dependent manner. Co-incubation of zinc with ZEA significantly reduced the ZEA-induced reactive oxygen species and malondialdehyde elevation by promoting the transcription of Mtf1 and Mt2. Meanwhile, two different zincs inhibited the ZEA-induced loss of mitochondrial membrane potential and elevation of late-stage apoptosis via activating the mitochondrial apoptotic pathway by recovering the mRNA and protein expression of pro-apoptotic genes (Bax, Casp3, Casp9). Zinc also recovered cells from S-phase cell cycle arrest. In addition, both of them promoted the ZEA-induced estrogen production but regulated the expression of steroidogenic enzymes (Star, Cyp11a1, Hsd3b1, Cyp17a1) in different way. All these results indicated that zinc could inhibit the reproductive toxicity of ZEA.

ZEA, a mycotoxin produced by several species of *Fusariu*m, is commonly found as a contaminant in cereals. Animals and humans are exposed to ZEA by consuming cereals and their by-products. Reproductive system is the main target of this mycotoxin, many previous studies have evidenced that both male and female fertility are injured by ZEA and its derivatives. For example, Benzoni *et al.* used direct measures of sperm integrity to show the potential adverse effect of ZEA exposure on boar fertility and discovered that alpha-Zearalenol and ZEA, at picomolar levels, negatively influenced chromatin structure stability and viability, respectively, whereas beta-Zearalenol negatively influenced the sperm motility at micromolar levels^1^. Minervini *et al.* demonstrate a negative effect of ZEA and its derivatives on meiotic progression of bovine oocytes by induced dose-dependent oocyte maturation delay and chromatin abnormalities at levels ranging from 0.3 to 30 μg/ml^2^. ZEA even delayed the in-utero development of rats, Collins *et al.* found that the number of viable fetuses, the fetal body weight and the maturity of skeleton were all significantly decreased after gavaging ZEA at a dose of 4 or 8 mg/kg to pregnant female Sprague Dawley (SD) rats^3^.

Due to its widespread threat to health, the detoxification of ZEA has been of major interest to researchers. Physical, chemical and biological methods have been developed to reduce and/or eliminate the toxic effects of contaminated products, improve food safety and minimize economic losses^4^. However, the process of detoxification is often accompanied by a loss of palatability and nutritional value of the food crop. The addition of nutrients to contaminated foods is one approach that reduces the toxicity of mycotoxins. Antioxidants, such as N-acetyl cysteine, vitamins C and E, have been reported to protect animals and cultured cells against the toxic effects of the T-2 toxin and ZEA^5^,^6^,^7^.

Zinc, as an essential element, binds to 10% of proteins in the mammalian proteome and is a cofactor for over 300 enzymes and more than 2,000 transcription factors. Zinc deficiency causes growth retardation, immune dysfunction, cognitive impairment, metabolic disorders, and infertility^8^. Zinc plays vital roles in the synthesis and metabolism of many types of hormones *in vivo*. Kralik *et al.* proved that zinc deficiency decreased the concentrations of triiodothyronine (T3) and free thyroxine (fT4) in serum by approximately 30% compared with zinc-adequate controls in SD rats, which indicated that zinc influences the levels of thyroid hormones^9^. Other studies have also shown that growth hormones and thymic hormones were affected by zinc supplementation or zinc deficiency^10^,^11^.

It has also been shown that the addition of zinc in feed was beneficial for animal reproduction and development. supplementation zinc in an organic form at the recommended level (300 mg of Zn/kg) for cows increased the milk yield and immunity^12^. The addition of zinc (800 ppm ZnCl_2_) inhibited the reduction of testis weight, the decrease of the epididymal sperm number and the remarkable degenerative lesions on the seminiferous tubules induced by low-dose mercury in male CD-1 mice^13^. Maternal zinc deficiency during pregnancy produced effects ranging from infertility, to embryonic/fetal death, to intrauterine growth retardation and teratogenesis^14^. Moreover, Batra *et al.* observed that zinc has protective effects against heavy metal-induced testis damage in male Portan rats^13^ due to its potential antioxidant properties. However, the potential for zinc to inhibit the reproductive toxicity of ZEA and the potential mechanisms are still unclear.

Due to the involvement of zinc in improving reproduction and the protective effects of zinc against several toxins, such as H_2_O_2_, heavy metals and ethanol^15^,^16^,^17^, we examined the effects of zinc on ZEA-induced reproductive toxicity. Because the ovary is a major target of ZEA toxicity, we employed immortalized murine ovarian granular KK-1 cells as an *in vitro* model to investigate the influence of zinc supplementation on the ZEA-induced reproductive damage at the molecular level. The present study is the first to explore both the protective effects of two sources of zinc and their mechanism of action in KK-1 cells from following aspects, including the cellular zinc response and homeostasis, oxidative stress, cell apoptosis and steroidogenesis.

## Results

### Zinc supplementation and zinc depletion both reduced the KK-1 cell viability

**Figure 1:**
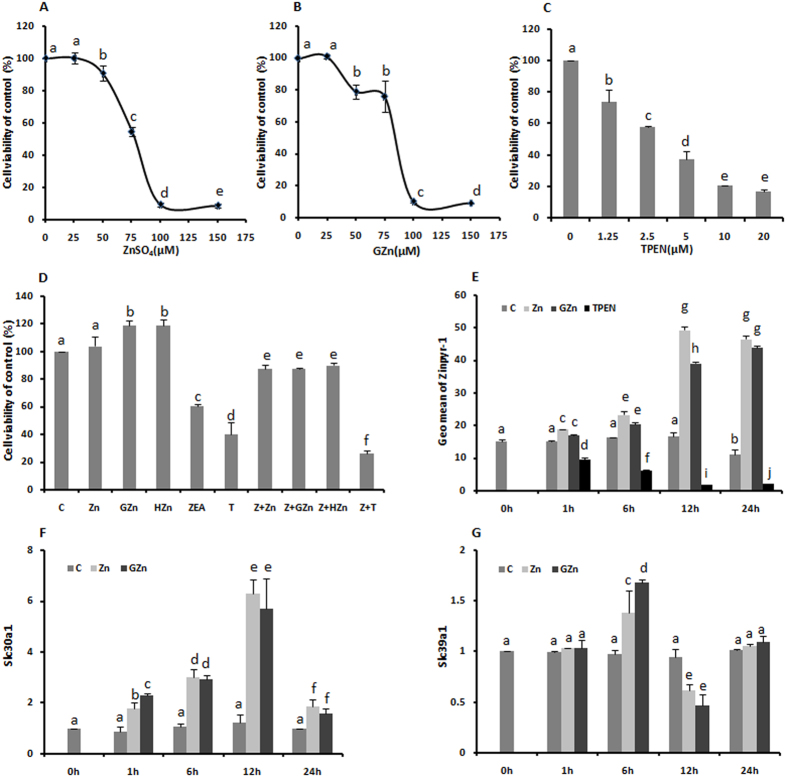
Cytotoxicity of ZnSO_4_, GZn, and TPEN. KK-1 cells were incubated with various concentrations of ZnSO_4_ (0–150 μM), GZn (0–150 μM), or TPEN (0–20 μM) for 24 h. The effects of ZnSO_4_ (**A**), GZn (**B**) and TPEN (**C**) on the cell viability were assessed by a CCK-8 assay. The effects of a 24-h coincubation with zinc and ZEA on the cell viability (**D**): C, 0.05% DMSO (v/v in DMEM); Zn, 25 μM ZnSO_4_; GZn, 25 μM GZn; HZn, 12.5 μM ZnSO_4_ and 12.5 μM GZn; ZEA, 20 μM ZEA; T, 2.5 μM TPEN; Z + Zn, 25 μM ZnSO_4_ and 20 μM ZEA; Z + GZn, 25 μM GZn and 20 μM ZEA; HZn + ZEA, 12.5 μM ZnSO_4_, 12.5 μM GZn and 20 μM ZEA; Z + T, 20 μM ZEA and 2.5 μM TPEN. The changes in the intracellular zinc concentration after treatment with ZnSO_4_, GZn or TPEN treatments (**E**). KK-1 cells were incubated with 0.05% DMSO (v/v in DMEM), 25 μM ZnSO_4_, 25 μM GZn or 2.5 μM TPEN. The cellular zinc concentration was determined using the FACSCalibur instrument at different time points (0, 1, 6, 12, 24 h) as described in the Materials and methods. The effects of ZnSO_4_ and GZn on the mRNA expression of zinc transporters Slc30a1 (**F**) and Slc39a1 (**G**). KK-1 cells were incubated with 0.05% DMSO (v/v in DMEM), 25 μM ZnSO_4_ or 25 μM GZn for different time periods (0, 1, 6, 12, 24 h). The values are the means ± SD of three independent experiments. The characters indicate significant differences between the compared groups (*p* < 0.05).

Because zinc and zinc chelator, N,N,N′N′-tetrakis (2-pyridylmethyl) ethylenediamine (TPEN), can both be toxic to cells, we determined the optimal concentrations of these substances for subsequent experiments by performing a CCK8 assay. Growing cultures of KK-1 cells were exposed to increasing concentrations of zinc sulfate (ZnSO_4_) (from 0 to 150 μM), zinc gluconate (GZn) (from 0 to 150 μM) and TPEN, a specific zinc chelator, (from 0 to 20 μM) for 24 h. All of the treatments caused a decrease in the cell viability in a dose-dependent manner (Fig. 1A–C), which indicated that both zinc overload and zinc depletion induce the death of KK-1 cells. Therefore, we choose the maximal non-toxic concentration of 25 μM ZnSO_4_, 25 μM GZn and near the IC_50_ concentration (2.5 μM) of TPEN for further studies. As shown in Fig. 1D, the results of co-incubation indicated that 25 μM of the two sources zinc addition could significantly prevent the decrease in cell viability induced by ZEA. We chose to use 20 μM ZEA, which was a concentration slightly lower than the IC_50_, based on the previous results of CCK8 studies (data not shown) and the report by Li *et al.*^18^.

### Both sources of zinc increased the intracellular zinc level in a time-dependent manner

To assess the relationship between zinc homeostasis and organic or inorganic zinc exposure, we used Zinpyr-1, a new membrane-permeant fluorescent sensor for Zn^2+^, to effectively label Zn^2+^in living cultured KK-1 cells. The uptake of Zinpyr-1 by cells is proportional to the zinc ion concentration. Figure 1E shows the findings in cells loaded with 25 μM ZnSO_4_, 25 μM GZn or 2.5 μM TPEN for different periods of time (1, 6, 12, and 24 h). The zinc fluorescence only decreased after 24 h of culture in the control, indicating that the fluorescence was stable over time. Both sources of zinc increased the intracellular zinc fluorescence in a time-dependent manner, and the changes showed similar tendencies. A 1-h treatment made no significant difference compared with the control, but after a 6-h treatment, the zinc fluorescence increased markedly, and it reached the peak value, which were 2.97 and 2.35-fold that observed in control cells, after another 6 h in culture. Between 12 h and 24 h, the zinc fluorescence decreased slightly, but remained high. Compared to the zinc supplementation groups, TPEN treatment decreased the zinc fluorescence from the start, with cultures beginning to show a significant reduction after 1-h of incubation.

We next evaluated the effects of the two types of zinc supplements on the transcription levels of the major zinc transport proteins, Slc30a1 and Slc39a1, by RT-PCR. We treated KK-1 cells with 25 μM ZnSO_4_ and 25 μM GZn for different lengths of time (1, 6, 12 and 24 h) (Fig. 1F,G), and found that the transcriptional level of Slc30a1 had increased dramatically by 6 h after the addition of 25 μM zinc (Fig. 1F). The Slc30a1 transcription level reached the peak values of 6.29- and 5.71-fold higher than the control (*p* < 0.05) after 12 h of treatment with ZnSO_4_ and GZn, respectively, and it decreased and returned to the basal level after 24 h. This was similar to the previously reported zinc response of HeLa cells^19^. These results confirmed that zinc treatment induced Slc30a1 transcription. Of note, after 6 h of incubation, the transcription level of Slc39a1 had increased to peak values of 1.39- and 1.68-fold higher compared to the control (*p* < 0.05), but the level suddenly decreased below the basal level after 12 h and returned to the basal level after 24 h. This was likely due to the zinc ions aggregating at approximately 12 h. Based on these findings, we speculated that KK-1 cells reached a new zinc homeostasis as the intracellular zinc concentration increased, without any apparent effects on the cell viability after 24 h zinc supplementation.

### Both sources of zinc protected KK-1 cells from oxidative damage

**Figure 2:**
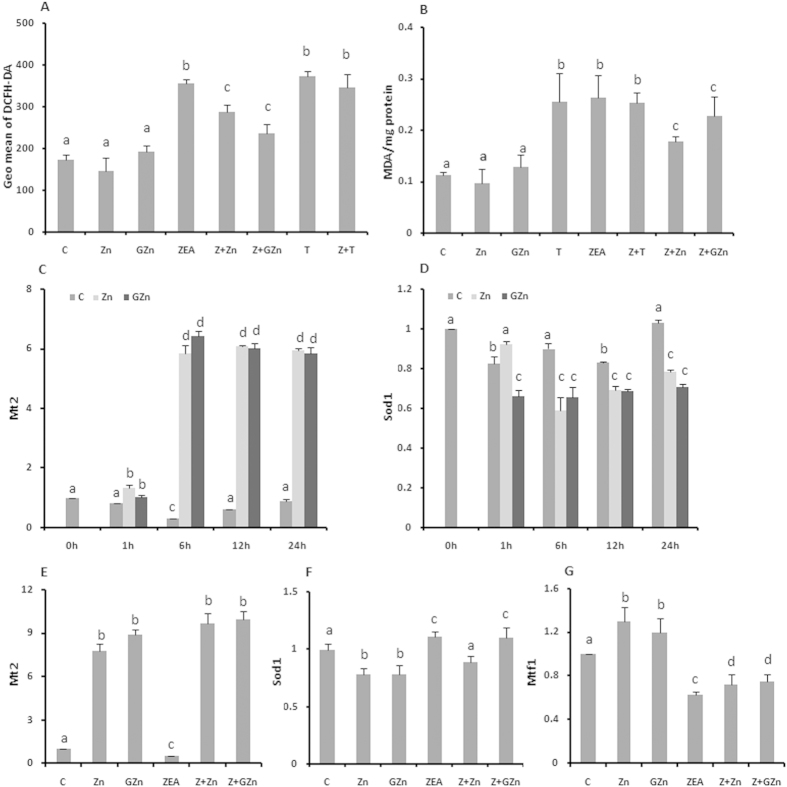
Zinc inhibits ZEA-induced oxidative stress. The effects of ZnSO_4_, GZn and TPEN on ZEA-induced ROS production (**A**) and the MDA increase (**B**). The ROS production is expressed by the fluorescence intensity of DCFH-DA, while the level of MDA is expressed as the ratio to the protein concentration. The effects of 25 μM ZnSO_4_ and GZn on the mRNA expression of Mt2 (**C**) and Sod1 (**E**) at different time points (0, 1, 6, 12, 24 h). The effects of 24 h of treatment with ZnSO_4_, GZn, or ZEA on the Mt2 (**D**), Sod1 (**F**) and Mtf1 (**G**) levels were also examined. KK-1 cells were treated as described in the legend for Fig. 1. The gene expression was assessed by real-time PCR after RNA isolation and reverse transcription. The cycle threshold (Ct) values of triplicate samples were averaged, and the mRNA expression relative to the control group was normalized to that of the housekeeping gene. The values are the means ± SD of three independent experiments. Different characters indicate that there was a significant difference between the compared groups (*p* < 0.05).

ROS generation was investigated using the 2′, 7′-dichlorodihydro fluorescein diacetate (DCFH-DA) fluorescent probe, which detects peroxide radicals and various other active oxygen radicals. Upon interaction with ROS, the DCFH-DA is converted to fluorescent DCF. Therefore, the level of intracellular ROS can be evaluated by analyzing the fluorescence of DCF. As shown in Fig. 2A, the level of intracellular ROS notably increased with the addition of ZEA, and zinc supplementation significantly reduced the ZEA-induced ROS production. Zheng *et al.* also showed that zinc ions could protect cells from ROS damage^20^. There were no significant differences between the cells treated with inorganic zinc and organic zinc. Lipid peroxidation was measured by determining the MDA production (Fig. 2B). Similar to the ROS generation, zinc supplementation significantly reduced the ZEA-induced MDA production, also with no significant differences noted between inorganic and organic zinc.

The transcription of Mtf1, Mt2 and Sod1 was examined to clarify the effects of zinc and ZEA treatment (Fig. 2). The KK-1 cells were first treated with 25 μM ZnSO_4_ and 25 μM GZn for different time period (1, 6, 12 and 24 h). The Mt2 mRNA level increased dramatically during the period from 1 h to 6 h after zinc treatment, reaching levels of 19.50- and 21.43-fold higher for ZnSO_4_ and GZn, respectively, than the corresponding levels in the control. On the contrary, the transcription level of Sod1 decreased by nearly 30% compared to the control in response to zinc treatment, and the time course had no effect on the Sod1 mRNA level. Second, it was observed that the exposure of KK-1 cells to 20 μM ZEA decreased the mRNA expression of Mt2 to half and the level of Mtf1 to 63% of the control, it also increased the mRNA expression of Sod1, although the change was not significant. The addition of both sources of zinc apparently inhibited the decrease in the mRNA expression of Mt2 and Mtf1 and slightly reduced the expression of Sod1 induced by ZEA. There were no differences between the two sources of zinc with regard to the inhibition of ZEA-induced oxidative stress.

### The two sources of zinc protect KK-1 cells from apoptosis

**Figure 3:**
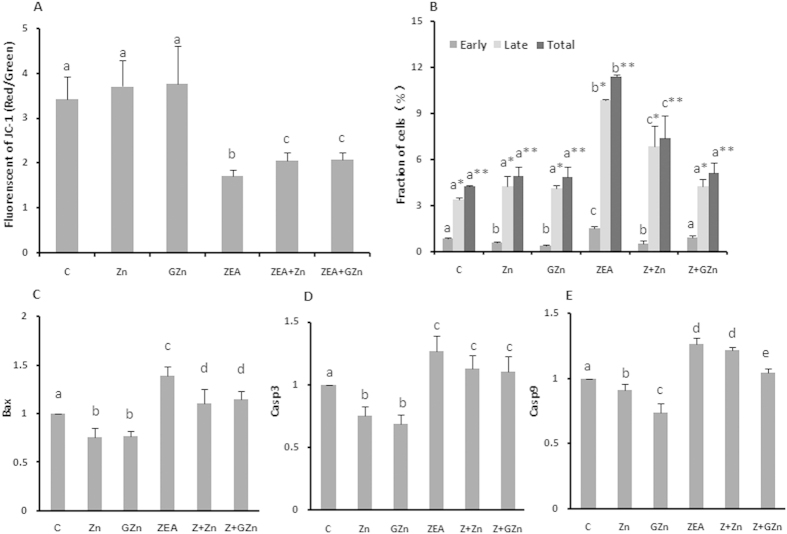
Zinc inhibits ZEA-induced cell apoptosis. The effects of ZnSO_4_, GZn, and ZEA on the Δψm (**A**) and the percentage of cell apoptosis (**B**). The Δψm is expressed as the fluorescence intensity ratio of the red over the green staining. The effects of ZnSO_4_, GZn, and ZEA on the mRNA expression of Bax (**C**), Casp3 (**D**) and Casp9 (**E**). KK-1 cells were treated as described in the legend for Fig. 1. The values are the means ± SD of three independent experiments. Different characters indicate significant differences between the compared groups (*p* < 0.05).

As shown in Fig. 3A, both types of zinc significantly reversed the ZEA-induced loss of mitochondrial membrane potential (Δψm). In detail, a marked decrease in the Δψm by approximately 50% occurred when cells were treated with 20 μM ZEA. However, co-incubation with 25 μM ZnSO_4_ or GZn for 24 h rescued the ZEA-induced Δψm loss by 19.88% and 21.20%, respectively, compared to the ZEA group. There was no difference between the effects of the two types of zinc. A decrease in the Δψm is an early indicator of cell apoptosis^19^, so we also tested the effects of zinc supplementation on the KK-1 cells by comparing the apoptosis rates. As shown in Fig. 3B, most of the cells were in the late stage of apoptosis. The rate was 2.84% in the control group, and increased to 8.78% in the ZEA group, which was reduced to 5.43% and 4.09% in the Z + Zn and Z + GZn groups, respectively (*p* < 0.05). The total cell death, including early apoptotic and late apoptotic cells, showed the same tendency.

**Figure 4:**
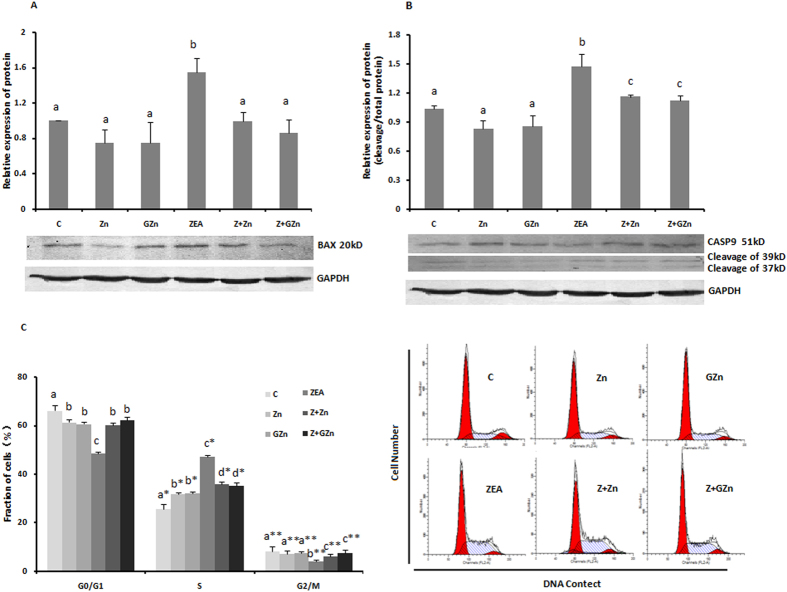
The effects of zinc were validated at the protein level by Western blotting. The effects of ZnSO_4,_ GZn, and ZEA on the protein expression of Bax (**A**) and Casp9 (**B**). The results of the Western blot analysis and the relative expression of each protein are shown. The effects of ZnSO_4_, GZn, and ZEA on the cell cycle was detected by a FACSCalibur instrument. The results are shown in (**C**). KK-1 cells were treated as described in the legend for Fig. 1. The values are the means ± SD of three independent experiments. Different characters indicate that there was a significant difference between the compared groups (*p* < 0.05).

All of the above results indicated that the addition of zinc efficiently protected KK-1 cells from apoptosis. To confirm this finding, we accumulated more evidence by performing a RT-PCR analysis of the expression of three pro-apoptotic genes: Bax, Casp9 and Casp3. Two sources of zinc inhibited the increased transcription levels of Bax (Fig. 3C), Casp3 (Fig. 3D) and Casp9 (Fig. 3E). Western blotting shown that both inorganic and organic zinc could down-regulate the expression of BAX and inhibit the activation of CASP9 (Fig. 4A,B). On the other hand, although adding zinc alone could reduce the level of Casp3 mRNA, co-incubation with zinc could not significantly prevent the ZEA-induced increase in expression (Fig. 3D). In summary, zinc exerted cytoprotective effects against ZEA-induced apoptosis, and GZn was better than ZnSO_4_ in inhibiting the Casp9 expression and late-stage apoptosis.

### Zinc supplementation prevents the ZEA-induced S-phase arrest

The cell cycle distribution was examined by flow cytometry. As shown in Fig. 4C, the treatment with ZEA increased the number of cells in the S phase from 25.70% to 47.26%, and decreased the number of cells in the G0/G1 and G2/M phases from 66.10% and 8.2% to 48.59% and 4.15%, respectively. In addition, co-incubation of ZnSO_4_ and GZn with ZEA reduced the percentage of cells in the S phase to 35.95% and 35.24%, whereas the number of cells in the G0/G1 phases increased to 60.12% and 62.24%, and those in the G2/M phases increased to 6.18% and 7.60%, respectively. Zinc supplementation could significantly prevent the ZEA-induced S-phase arrest, with no significant differences noted between organic zinc and inorganic zinc.

### Both sources of zinc affect the steroidogenesis in KK-1 cells

**Figure 5:**
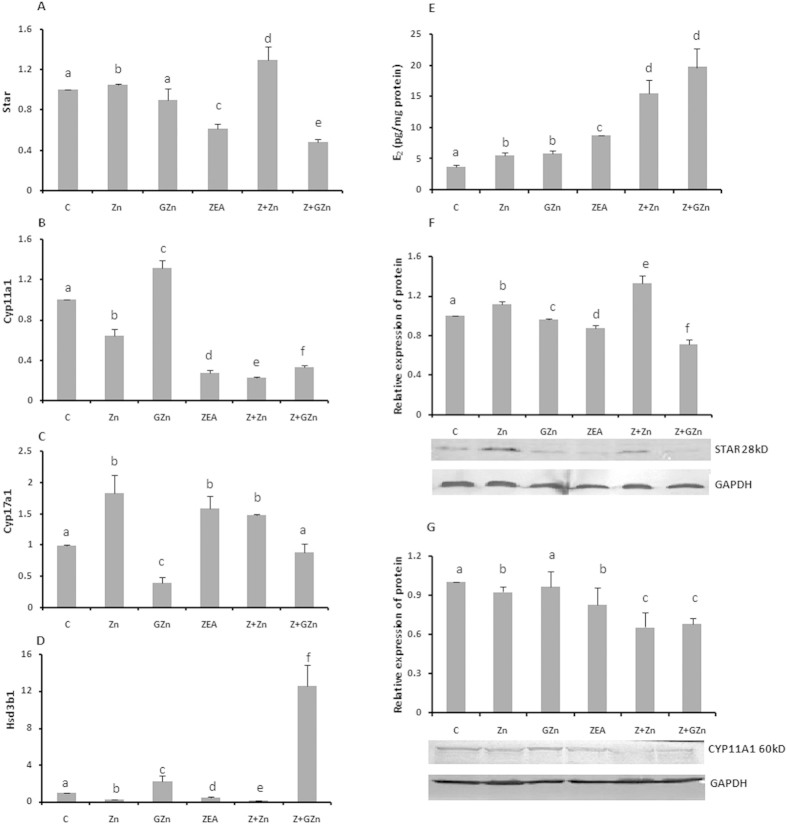
Zinc regulates steroidogenic enzymes and promotes estrogen production. The effects of ZnSO_4_, GZn, and ZEA on the transcription of Star (**A**), Cyp11a1 (**B**), Cyp17a1 (**C**) and Hsd3b1 (**D**) are shown. The effects of ZnSO_4_, GZn, and ZEA on the protein expression of these steroidogenic enzymes were determined by Western blotting, and the results and relative expression of Star (**F**) and Cyp11a1 (**G**) are shown. The effects of ZnSO_4_, GZn, and ZEA on the estrogen production were determined by RIA (**E**). KK-1 cells were treated as described in the legend for Fig. 1. The values are the means ± SD of three independent experiments. Different characters indicate significant differences between the compared groups (*p* < 0.05).

As a type of reproductive cell, KK-1 cells are unique because they have the ability to synthesize steroid hormones. We therefore assessed the effects of the two sources of zinc on the estrogen production by a radioimmunosorbent assay (RIA) (Fig. 5E). Both zinc and ZEA could promote estrogen production, with levels of 5.51, 5.87, and 8.71 pg/mg protein respectively, in the ZnSO_4_-, GZn- and ZEA-treated groups in comparison to the control level of 3.72 pg/mg protein. Moreover, co-incubation with these zinc sources and ZEA led to synergistic increases to 15.53 and 19.72 pg/mg protein, respectively.

In addition to evaluating the hormone synthesis, we also examined the mRNA levels of four important genes encoding steroidogenic enzymes required for progesterone or estrogen production in KK-1 cells (Star, Cyp11a1, Cyp17a1, and Hsd3b1)in the different groups (Fig. 5A–D). We validated the expression of Star and Cyp11a1 at the protein level by Western blotting (Fig. 5F,G). Interestingly, 25 μM ZnSO_4_ and 25 μM GZn affected the expression levels of all four enzymes, but in different ways (Table 1). Co-incubation with ZnSO_4_ mainly increased the mRNA and protein levels of Star while co-incubation with GZn elevated the mRNA levels of Cyp11a1,Cyp17a1and Hsd3b1. The mRNA and protein expression of Star and Cyp11a1 displayed different trends. This may have resulted from the delayed induction of enzyme synthesis^21^. In the early 1990s, Hanukoglu *et al.* found that brief (30 min) stimulation of adrenocortical cells with adrenocorticotropin (ACTH) led to both a rapid increase in steroid secretion and a delayed increase in enzyme synthesis that peaked 36 h later^22^. The above results indicated that both of the sources of zinc promoted estrogen production, but they influenced the steroidogenic enzyme levels in different ways.

## Discussion

The effects of ZEA-induced reproductive toxicity have been widely investigated *in vivo*, especially in sensitive animal species, such as swine^23^. Likely because of the multiple interactions that occur in whole organisms during ZEA exposure, the results of *in vitro* studies may only be partial agreement with those of *in vivo* studies, but cell-specific responses can only be understood from *in vitro* investigations. Moreover, *in vitro* experiments may contribute to understanding the direct biological effects of ZEA and defining its mechanism of action, which would help understand the effects of ZEA on the testes and ovaries^24^,^25^. Thus in the present study, cultured KK-1 cells were employed to explore the protection mechanism of two distinct sources of zinc on ZEA-induced reproductive toxicity *in vitro*.

Oxidative stress, inducing an imbalance between the level of ROS and the cellular defense system^26^,^27^, has been proposed as a possible mechanism of reproductive damage in both human and animals^28^. Municipal landfill leachate induced the reproductive toxicity to male rats by increased sperm abnormalities and disrupted the antioxidant systems of rats sperm with concomitant elevation in hydrogen peroxide and malondialdehyde levels^29^. The cyclophosphamide -treated group showed enhancement of lipid peroxidation leading to testicular reproductive toxicity while total flavonoids of epimedium restored these oxidative damages by up-regulating the expression of antioxidant enzymes, especially SOD3 and GPX1^30^. Yang *et al.* illustrated that carbon disulfide directly induced DNA damage in endometrial cells and enhanced the action through oxidative stress, both of which were responsible for carbon disulfide-induced embryo loss in pregnant female mice^31^.

In this study, co-incubation of zinc with ZEA significantly reduced the ZEA-induced ROS generation and lipid peroxidation, indicating that zinc increased the antioxidant processes in KK-1 cells. Then, we explored the potential mechanism of action by detecting the mRNA levels of Mt2, Mtf1 and Sod1. Mt2, ubiquitous and stress-inducible in mice, encodes one type of metallothioneins which in favor of scavenging ROS^32^. As shown in Fig. 2, treatment with either of the two sources of zinc strongly increased the transcription level of Mt2, and zinc addition significantly inhibited the ZEA-induced decrease in Mt2. Mtf1 has been proven to be an essential cell stress responder following oxidative stress^33^. When cells are treated with heavy metals, it is activated to bind the metal response element^34^, which induces the transcription of target genes, notably metallothioneins, which have MRE binding sites in the 5′ regulatory region^15^,^35^. In our study, the expression of Mtf1 changed in the same way as Mt2, which indicated that zinc regulated the transcription of Mt2 through the activation of the Mtf1. However, zinc supplementation inversely decreased the expression of Sod1, and there was no apparent change in the expression in ZEA-treated cells following zinc supplementation (Fig. 2E,F). Zheng *et al.* also found that Sod1 was not influenced by ZnSO_4_ in HepG2 cells^20^. However, there has been a lot of evidence proving that zinc increased the activity of Sod1^36^. These results indicated that although zinc plays an important role in the function of Sod1, it has no obvious effects on the transcription of the gene.

In summary, zinc could enhance the resistance of KK-1 cells to the reproductive toxicity of ZEA by inhibiting the ZEA-induced oxidative stress via activation of the transcription expression of antioxidative genes: Mtf1 and Mt2.

Apoptosis is a highly regulated form of programmed cell death, which plays a key role in the development and homeostasis of reproductive systems *in vitro* and *vivo*. Ovarian granular cells, which grow around the mammalian oocytes in the ovary, can promote follicle growth and provide energy for the oocytes. *In vivo*, a high level of granular cell apoptosis will affect the normal maturation process of follicles, resulting in follicular atresia^37^. *In vitro*, many previous studies have demonstrated that xenobiotics could promote mouse Leydig or spermatogenic cells apoptosis which was one of the main mechanisms of their reproductive toxicity^38^,^39^. As an early event in apoptosis, mitochondrial dysfunction is always considered^40^. Chayma Bouaziz *et al.* reported that ZEA induced mitochondrial alterations in HepG2 cells, including a loss of the Δψm, PTPC opening, and cytochrome c release^41^. Guo *et al.* demonstrated that zinc deficiency in osteoblastic MC3T3-E1 cells induced cell apoptosis via the mitochondria-mediated pathway, resulting in a reduction of the Δψm and increased levels of BAX in the mitochondrial fraction and increased levels of cytochrome c, apoptosis-inducing factor (AIF), and cleaved of CASP3 and CASP9 in the cytosolic fraction^42^. Similarly, our present study showed that zinc supplementation inhibited the increase in late-stage apoptosis and the loss of Δψm in KK-1 cells, which had been induced by ZEA. The mRNA expression of pro-apoptotic genes including Bax, Casp3 and Casp9, were determined by RT-PCR (Fig. 3C–E). In addition, the BAX level decreased markedly, and the cleavage of CASP9 was inhibited after co-incubation. All of these results indicated that the two sources of zinc inhibited the apoptosis of KK-1 cells via the Caspase-dependent mitochondrial apoptotic pathway.

In a word, zinc could enhance the resistance of KK-1 cells to ZEA-induced reproductive toxicity by inhibiting the ZEA-induced apoptosis via regulating key genes in the Caspase-dependent mitochondrial apoptotic pathway. Moreover, the proliferation of a cell population is regulated by the balance among cell division, growth arrest, differentiation and programmed cell death^43^. Under our experimental conditions, both inorganic and organic zinc could inhibit the ZEA-induced S-phase cell cycle arrest. However, the exact mechanism leading to this effect will need to be elucidated in further studies.

In mice, the pathways of steroid hormone biosynthesis starts with cholesterol^44^. Trophic hormones (e.g., ACTH, luteinizing hormone (LH), and FSH) activate a chain of reactions that lead STAR to translocate cholesterol from the outer to the inner mitochondrial membrane, where it is converted to pregnenolone by CYP11A1, which is present in the inner mitochondrial membrane in all steroidogenic cells^45^. After this rate-limiting step, pregnenolone is converted to progesterone by HSD3B1 in the microsomal compartment. Progesterone is then partly converted to testosterone by CYP17A1 and HSD17B1. Finally, CYP19A1 converts testosterone to estradiol. However, in the ovary, CYP17A1 is expressed in the theca interna cells. Antibody and cDNA probes have shown very low expression of Cyp17a1 in the granulosa cells of humans and rats^46^,^47^,^48^. Thus, theca interna cells can synthesize androgens, but granulosa cells which produce estrogens are dependent on the androgen precursor supply from the theca interna. This process is called the two cell hypothesis of follicular estrogen production^49^. In our experiments, we added testosterone to activate the steroid hormone biosynthesis pathway and to serve as the substrate for estrogen synthesis.

In the present study, we investigated the effects of zinc on steroidogenic enzymes and estrogen production *in vitro* for the first time, and the results are described in section 3.6 (Results). In brief, we found that both zinc and ZEA could promote estrogen production, and co-incubation likely led to a synergistic effect. Previous studies have shown that adding exogenous 17β-estradiol may be protective against oxidative stress-induced damage and even apoptosis both *in vivo* and *in vitro*. For example, Wang *et al.* found that 17β-estradiol could ameliorate light-induced retinal damage in SD rats via its antioxidative effects, and they showed that its underlying mechanism involves the regulation of the gene expression levels of antioxidant enzymes (SOD, CAT, and GPX) and proteins (TRX and NRF2)^50^. Kanda *et al.* provided evidence that 17β-estradiol inhibits oxidative stress-induced apoptosis in keratinocytes by promoting Bcl2 expression^51^. Thus, we speculate that the slight increase in estrogen promoted by ZEA may have been a stress response to the ZEA-induced oxidative damage, and both sources of zinc enhanced this response. However, further investigations will be needed to confirm whether this is the case.

Generally, there are considered to be three types of zinc sources; inorganic zinc (e.g., zinc sulfate, zinc oxide and zinc chloride) and organic zinc which includes zinc salts of organic acids (e.g., zinc gluconate, zinc citrate, zinc acetate) and zinc chelates of amino acids or peptides (e.g., zinc methionine, lysine zinc)^52^. Different zinc sources have been widely used in animal nutrition studies to alleviate zinc deficiency.

Lin *et al.* evaluated zinc methionine (ZnMet), zinc lysine (ZnLys), zinc glycine (ZnGly) and ZnSO_4_ as dietary zinc sources for *Litopenaeus vannamei*. Their results showed that when the shrimp were fed diets with 6.5 mg Zn/kg diet, organic zinc supplementation produced significantly higher growth, survival, and immune parameters than ZnSO_4_ treatment. Shrimp supplemented with ZnMet had the highest weight gain and best immune parameters. However, there were no significant differences between the ZnLys and ZnGly groups. These results suggest that the zinc from ZnMet was a better source than the other zinc forms^53^.

The bioavailability of different zinc compounds (sulfate, gluconate and citrate) was also compared in the male rat prostate after adding three different doses (3.0, 15.0, and 50.0 mg Zn/kg b.w.) to their diet for 30 days. Only zinc gluconate and zinc citrate increased the zinc concentrations in the dorsolateral lobe of the prostate compared to controls^54^. In addition, Wegmüller *et al.* used the double-isotope tracer method with ^67^Zn and ^70^Zn, and found that the median (IQR) fractional absorption of zinc from zinc oxide (49.9%) was significantly lower than that from zinc citrate (61.3%) and zinc gluconate (60.9%), but there was no marked difference between two organic zinc sources^55^. However, few studies have focused on the differences between sources of zinc *in vitro*. Yu *et al.* investigated the effects of different zinc sources and levels on the inhibition of the thymocyte apoptosis induced by glucocorticoid *in vitro.* Zinc sulfate and zinc methionine were supplemented into the medium at levels of 0, 50, 100, 500 and 1000 μM. The intracellular calcium concentrations of the cells cultured with zinc methionine were higher than those cultured with zinc sulfate at the same levels, but both of them could modulate the glucocorticoid-induced apoptosis of thymocytes. The mechanism might involve the exchange of intracellular calcium or the redox of cells, and the two forms of zinc might have different effects on the regulation of these processes^56^.

Above all, we insured that zinc sulfate and zinc gluconate could inhibit the reproductive toxicity of ZEA on KK-1 cells.

## Methods

### Chemicals and reagents

Fetal bovine serum (FBS) was obtained from HyClone,USA; 100 U/ml penicillin, 100 μg/ml streptomycin, 250 ng/ml amphotericin B, high-glucose Dulbecco’s minimal essential medium (DMEM), 0.25% trypsin (w/v) and 0.52 mM EDTA were purchased from Macgene, PRC. ZnSO_4_·7H_2_O, GZn, follicle-stimulating hormone (FSH), testosterone (T) and the TPEN, were purchased from Sigma,USA.

### Cell cultured and treatments

KK-1 cells were cultured in DMEM supplemented with 10% FBS, 100 U/ml penicillin, 100 μg/ml streptomycin, and 250 ng/ml amphotericin B at 37 °C in a humidified 5% CO_2_ incubator (Sanyo, JP).

The cells were divided into different experimental groups according to the study requirements. The different agents used for the treatments were diluted with serum-free medium, and cells were treated for 24 h. The cells were dispersed with 0.25% trypsin (w/v) and 0.52 mM EDTA.

### Cell viability assay

Cell viability was determined using the Cell Counting Kit-8 (Beyotime, PRC) according to the manufacturer’s instructions. Briefly, 1 × 10^4^ cells/well were seeded in a 96-well plate and grown at 37 °C for 24 h, then were washed once with phosphate-buffered saline (PBS) after being treated in 5 technical replicates. Subsequently, 10 μl of WST-8 and 100 μl of PBS were added to each well, and the cells were incubated at 37 °C for 2 h. Finally, the dye absorbance was determined at 450 nm using a microplate reader (Thermo, USA).

### Zn^2+^ concentration determination

The intracellular Zn^2+^ level in the KK-1 cells was measured using the Zn^2+^ indicator, Zinpyr-1 (Sigma, USA). Cells were cultured in 6-well plates at a density of 2 × 10^5^ cells/well, grown at 37 °C for 24 h and treated in 3 technical replicates. Then, the cells were loaded with 12 mM Zinpyr-1 solution with serum-free medium and incubated at 37 °C for 20 min. Before detecting the fluorescence intensity using a FACSCalibur instrument (BD Biosciences, USA), the cells were rinsed twice with PBS to remove the fluorescent probe and were dispersed by trypsin. The emission was detected in the FL1. At least 1 × 10^4^ cells in the selected gate were collected for further analyses.

### ROS determination

ROS generation was measured using a fluorescent probe assay kit (Beyotime, PRC), which contained DCFH-DA. The protocol was as follows: Cells were cultured in 6-well plates at a density of 2 × 10^5^ cells/well, grown at 37 °C for 24 h, and treated in 3 technical replicates. The cells were then washed once with PBS and incubated for 30 min at 37 °C with 10 μM of the probe. Finally, before detecting the fluorescence intensity using the FACSCalibur instrument, the cells were rinsed twice with PBS and dispersed by trypsin. The fluorescence intensity in FL1 was proportional to the ROS production. At least 1 × 10^4^ cells in the selected gate were collected for further analyses.

### MDA assay

KK-1 cells were cultured at 6 × 10^5^ cells/ml and seeded onto 60 mm dishes. The cells were treated for 24 h and then harvested by trypsinization, followed by washing twice with PBS. Cell lysates were homogenized and centrifuged at 15,000 × g for 15 min at 4 °C after being lysed in RIPA buffer (Beyotime, PRC)

The MDA assay was performed with a Lipid Peroxidation Kit (Nanjing Jiancheng, PRC) following the protocol provided by the manufacturer. Briefly, 100 μl of supernatant was mixed with 1 ml of malondialdehyde (MDA) working solution and was boiled at 95 °C for 45 min. Samples were cooled down to room temperature in a water bath and then centrifuged at 1,000 × g for 10 min. The absorbance at 532 nm was measured in a 96-well plate (250 μl/well) with a microplate reader (Thermo, USA). The results of the MDA assay were expressed as micromoles of MDA per milligram of protein, which was measured using a BCA Kit (Beyotime, PRC)^57^.

### Δψm measurement

Δψm was measured using JC-1 dye. The cells were seeded on 6-well plates and exposed to different treatments. 24 h later, the cells were incubated with 5 μg/ml of JC-1 staining solution (Beyotime, PRC) for 20 min at 37 °C. After the cells were washed twice with the JC-1 staining buffer, the fluorescence densities of the JC-1 monomers (λex = 488 nm, λem = 529 nm) and JC-1 aggregates (λex = 524 nm, λem = 594 nm) were detected using a microplate reader. The Δψm of the KK-1 cells was expressed as the fluorescence intensity ratio of the JC-1 aggregates to that of the JC-1 monomers. Three technical replicates were carried out in each group.

### Cell apoptosis

Cellular apoptosis was determined using the Annexin V-FITC Apoptosis Detection Kit (Nanjing Jiancheng, PRC). According to the protocol, KK-1 cells were cultured at 6 × 10^5^ cells/ml and seeded onto 60 mm dishes. The cells were treated for 24 h and then harvested by trypsinization, followed by washing twice with cold PBS. Approximately 1 × 10^5^ to 1 × 10^6^ cells were resuspended in 200 μl of 1 × binding buffer and were transferred to a sterile flow cytometry glass tube. The cells were incubated with 5 μl Annexin V-FITC and 10 μl propidium iodide (PI) in the dark at room temperature. The apoptotic cells were identified and analyzed using a FACSCalibur instrument. The emission wavelengths of Annexin V-FITC and PI were detected in the FL1 and FL2 channels. The percentages of normal, early apoptotic, late apoptotic, and necrotic cells were calculated using the CellQuest software program.

### Cell cycle

A cell cycle analysis was conducted according to the method described by Cai *et al.*^58^ with slight modifications. Briefly, cells were cultured and synchronized before they were divided for six different experiments in three technical replicates, then were harvested in PBS-0.05% trypsin buffer. After centrifugation for 5 min at 1500 rpm at 4 °C, the supernatant was removed, and the pellet was resuspended in ice-cold 70% ethanol. Cells were digested with 2 mg/ml RNase A at 37 °C for 20 min and were stained with 50 g/ml PI containing 0.1% Triton X-100 and EDTA 0.02 mg/ml. Samples were analyzed for their DNA content and cell cycle distribution using a FACSCalibur flow cytometer with the ModFit software program.

### E_2_ determination by RIA

KK-1 cells were cultured at 1 × 10^5^ cells/ml and seeded onto 12-well plates. The cells were treated for 24 h, followed by washing twice with PBS. Then, 1 ml/well of serum-free medium containing 75 ng/ml of FSH and 5 μg/ml of T was added for another 4 h at 37 °C. Finally, the serum-free medium solution was collected from every well, and the level of E_2_ was measured following the manufacturer’s instructions for the E_2_ Kit (Beijing North Biological Institute, PRC) using a RIA method. The results were expressed as the micromoles of E_2_ per milligram of protein, which was measured using a BCA Kit.

### RNA extraction and RT-PCR

Total RNA was isolated from the cells using the TRIzol reagent (CWBio, PRC) according to the manufacturer’s instructions. The integrity of the purified RNA was tested on a 1% agarose gel and was quantified spectrophotometrically at 260 nm and 280 nm. First-strand cDNA was synthesized from 2 μg of RNA using the Reverse Transcription System (Promega, USA) according to the manufacturer’s protocol using oligo (dT) as the primers. The RT-PCR reaction was performed using the Real Master Mix (SYBRGreen, Tiangen, PRC) with 200 nM of forward primer, 200 nM of reverse primer (Supplemental Table 1), and 100 ng cDNA as the template. The RT-PCR was performed on a 7500 Real-Time PCR System (Applied Biosystems, USA) with the following conditions: 95 °C for 5 min and then 40 cycles of 95 °C for 30 s, 60 °C/64 °C for 30 s and 72 °C for 30 s. The GAPDH gene was selected as the control gene. The relative expression of each target gene, which was calculated using the average value of the ΔCt between the target gene and the control gene (GAPDH), was expressed as 2^−ΔΔCt^.

### Protein isolation and Western blotting

Following different treatments, the cells were lysed on ice with RIPA Lysis Buffer supplemented with 1 mM PMSF (Beyotime, PRC). Cells were then homogenized using a 1 ml syringe^59^,^60^. Cellular lysates were centrifuged at 13,000 × g for 10 min at 4 °C. The supernatant proteins were collected and quantified using a BCA Kit. Equal amounts (40 μg) of protein from each sample were loaded on 13% SDS-PAGE gels and blotted on nitrocellulose membranes for 1.5 h at 80 V. Nonspecific binding was blocked by incubating the samples in blocking buffer (1% BAS and Tris-buffered saline containing 0.1% Tween-20 (TBST)) for 1 h, and the membranes were incubated for 1 h with one of the following primary antibodies: rabbit anti-GAPDH (Cell Signaling, 4970; 1:1000), rabbit anti-BAX (Cell Signaling, 2772; 1:1000), mouse anti-CASP9 (Cell Signaling, 9508; 1:1000), rabbit anti-STAR (Cell Signaling, 8449; 1:1000), or rabbit anti-CYP11A1 (Santa Cruz, 292456; 1:1000).

### Statistics

The data were expressed as the means ± standard deviation. The experiments were repeated at least twice, and each experiment included at least triplicate treatments. The data from different treatments were subjected to an analysis of variance (ANOVA), and the comparisons of the means were performed using Duncan’s multiple range test. All of the statistical analyses were performed using the SPSS 16.0 software program. In all figures, the characters above the error bar indicate that there are significant differences between the compared groups (*p* < 0.05).

## Additional Information

**How to cite this article**: Li, Y. *et al.* Zinc inhibits the reproductive toxicity of Zearalenone in immortalized murine ovarian granular KK-1 cells. *Sci. Rep.* **5**, 14277; doi: 10.1038/srep14277 (2015).

## Supplementary Material

Supplementary Information

## Figures and Tables

**Table 1:** The influence of different treatments on the mRNA expression of steroidogenic enzyme.

	Star	Cyp11a1	Hsd3b1	Cyp17a1
20 μM ZEA	—	—	—	+
25 μM ZnSO_4_	↑+	—	—	+
25 μM GZn	— —	↑+	↑+	↑—

**Notes:** **— —** no significant difference, — significant down,  + significant up, **↑**significant inhibit.
