# Supplementary material for: Zinc inhibits the reproductive toxicity of Zearalenone in immortalized murine ovarian granular KK-1 cells
Source: Sci Rep. 2015 Sep 23;5:14277. doi: 10.1038/srep14277 (PMC4585791; doi:10.1038/srep14277)
Supplement: Supplementary Information [file srep14277-s1.pdf]

Title: Zinc inhibits the reproductive toxicity of Zearalenone in immortalized murine ovarian granular KK-1 cells

Author List: Yijia Li, Xiaoyun He, Xuan Yang, Kunlun Huang, Yunbo Luo, Liye Zhu, Yuzhe Li, Wentao Xu

Supplementary Table S1: Primers used in quantitative real-time PCR

Specificity	Sequence(5'-3')	Product(bp)	Reference
GAPDH	F-AAGGACACTGAGCAAGAG	147	Li et al. [1]
	R- CGAACTTTATTGATGGTATTC		
Slc30a1	F-AAAATTGAGAAGTGATGACC	195	Designed
	R- CCCTTCCAGTTAAAGTAAAA		
Slc39a1	F- GACAGCAATGGAGTGAGAC	210	Designed
	R-AGGCTGCAGATGAGTGT		
Mt2	F- CTCCTAGAACTCTTCAAACC	141	Designed
	R- GAAGTACATTTGCATTGTTT		
Mtf1	F- TCTTTAGGCAAGGTATTTAGG	123	Designed
	R- TCAGAAATTCATACTCCTGGT		
Sod1	F- GTCCATTGAAGATCGTGT	128	Designed
	R- CATTTCCAGTCTTTGTACTTT		
Bax	F- CTAGCAAACTGGTGCTCAAGG	147	Designed
	R- CGAAGTAGGAGAGGAGGCCT		
Casp3	F-AGGGGTCATTTATGGGACA	422	Designed
	R- TACACGGGATCTGTTTCTTTG		
Casp9	F- GTACATCGAGACCTTGGAT	198	Designed
	R- GAGAATAATGAGGCAGAGAG		
Star	F-TGTCAAGGAGATCAAGGTCCTG	334	Li et al. [1]
	R- CGATAGGACCTGGTTGATGAT		
Cyp11a1	F-CGACTCCTCAGAACTAAGAC	143	Li et al. [1]
	R-CTATAAAGGACACCAGGGTA		
Hsd3b1	F-ACTGCAGGAGGTCAGAGCT	565	Li et al. [1]
	R-GCCAGTAACACACAGAATACC		
Cyp17a1	F-ATCTGTCTACGCTCATCTTC	120	Li et al. [1]
	R-CAATACCCTCTGTAAAGGTC		

[1] Li, Y. et al. Mitochondrial proteomic analysis reveals the molecular mechanisms underlying reproductive toxicity of zearalenone in MLTC-1 cells. Toxicology. 324, 55-67(2014).

Supplementary Figure S1 Cell apoptosis results from flow cytometry

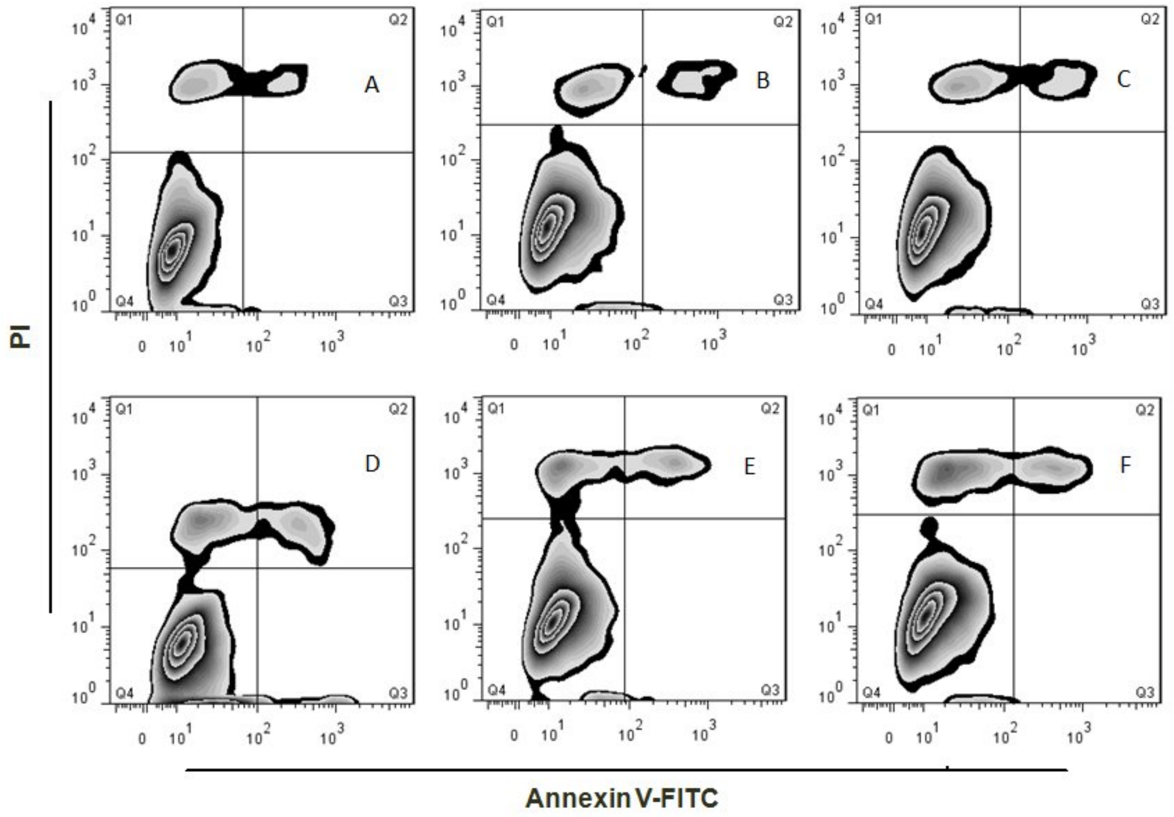

Supplementary Figure S1: The flow cytometry results of cell apoptosis with different treatments. C(A),Zn(B),GZn(C),ZEA(D),Z+Zn(E),Z+GZn(F).

Figure legends

Fig. 1. Cytotoxicity of $ZnSO_4$, GZn, and TPEN. KK-1 cells were incubated with various concentrations of $ZnSO_4$ (0-150 μM), GZn (0-150 μM), or TPEN (0-20μM) for 24 h. The effects of $ZnSO_4$ (A), GZn (B) and TPEN (C) on the cell viability were assessed by a CCK-8 assay. The effects of a 24-h coincubation with zinc and ZEA on the cell viability (D): C, 0.05% DMSO (v/v in DMEM); Zn, 25 μM $ZnSO_4$; GZn, 25 μM GZn; HZn, 12.5 μM $ZnSO_4$ and 12.5 μM GZn; ZEA, 20 μM ZEA; T, 2.5 μM TPEN; Z+Zn, 25 μM $ZnSO_4$ and 20 μM ZEA; Z+GZn, 25 μM GZn and 20 μM ZEA; HZn+ZEA, 12.5 μM $ZnSO_4$, 12.5 μM GZn and 20 μM ZEA; Z+T, 20 μM ZEA and 2.5 μM TPEN. The changes in the intracellular zinc concentration after treatment with $ZnSO_4$, GZn or TPEN treatments (E). KK-1 cells were incubated with 0.05% DMSO (v/v in DMEM), 25 μM $ZnSO_4$, 25 μM GZn or 2.5 μM TPEN. The cellular zinc concentration was determined using the FACS Calibur instrument at different time points (0, 1, 6, 12, 24 h) as described in the Materials and methods. The effects of $ZnSO_4$ and GZn on the mRNA expression of zinc transporters Slc30a1 (F) and Slc39a1 (G). KK-1 cells were incubated with 0.05% DMSO (v/v in DMEM), 25 μM $ZnSO_4$ or 25 μM GZn for different time periods (0, 1, 6, 12, 24 h). The values are the means ± SD of three independent experiments. The characters indicate significant differences

between the compared groups ($p < 0.05$).

Fig. 2. Zinc inhibits ZEA-induced oxidative stress. The effects of $ZnSO_4$, GZn and TPEN on ZEA-induced (A):ROS production (A) (B):and the MDA increaselevel (B). The ROS production is expressed by the fluorescence intensity of DCFH-DA, while the level of MDA is expressed as the ratio to the protein concentration. The effects of 25 μM $ZnSO_4$ and GZn on the mRNA expression of Mt2 (C) and Sod1 (E) at different time points (0, 1, 6, 12, 24 h). The effects of 24 h of treatment with $ZnSO_4$, GZn, or ZEA on the Mt2 (D), Sod1 (F) and Mtf1 (G) levels were also examined. KK-1 cells were treated as described in the legend for Fig. 1. The gene expression was assessed by real-time PCR after RNA isolation and reverse transcription. The cycle threshold (Ct) values of triplicate samples were averaged, and the mRNA expression relative to the control group was normalized to that of the housekeeping gene. The values are the means ± SD of three independent experiments. Different characters indicate that there was a significant difference between the compared groups ($p < 0.05$).

Fig. 3. Zinc inhibits ZEA-induced cell apoptosis. The effects of $ZnSO_4$, GZn, and ZEA on the $\Delta\psi m$ (A) and the percentage of cell apoptosis (B). The $\Delta\psi m$ is expressed as the fluorescence intensity ratio of the red over the green staining. The effects of $ZnSO_4$, GZn, and ZEA on the mRNA expression of Bax (C), Casp3 (D) and Casp9 (E). KK-1 cells were treated as described in the legend for Fig. 1. The values are the means ± SD of three independent experiments. Different characters indicate significant differences between the compared groups ($p < 0.05$).

Fig. 4. The effects of zinc were validated at the protein level by Western blotting. The effects of $ZnSO_{4,}$ GZn, and ZEA on the protein expression of Bax (A) and Casp9 (B). The results of the Western blot analysis and the relative expression of each protein are shown. The effects of $ZnSO_4$, GZn, and ZEA on the cell cycle was detected by a FACS Calibur instrument. The results are shown in (C). KK-1 cells were treated as described in the legend for Fig. 1. The values are the means ± SD of three independent experiments. Different characters indicate that there was a significant difference between the compared groups ($p < 0.05$).

Fig. 5. Zinc regulates steroidogenic enzymes and promotes estrogen production. The effects of $ZnSO_4$, GZn, and ZEA on the transcription of Star (A), Cyp11a1 (B), Cyp17a1 (C) and Hsd3b1(D) are shown. The effects of $ZnSO_4$, GZn, and ZEA on the protein expression of these steroidogenic enzymes were determined by Western blotting, and the results and relative expression of Star (F) and Cyp11a1 (G) are shown. The effects of $ZnSO_4$, GZn, and ZEA on the estrogen production were determined by RIA (E). KK-1 cells were treated as described in the legend for Fig. 1. The values are the means ± SD of three independent experiments. Different characters indicate significant differences between the compared groups ($p < 0.05$).
